# Predicting risks of physical health deterioration in a place of safety

**DOI:** 10.1192/bjo.2021.81

**Published:** 2021-06-18

**Authors:** Alex Berry, Florence Dalton, Michael Dunning, Freddie Johansson

**Affiliations:** 1 National Hospital for Neurology and Neurosurgery; 2 Camden and Islington Foundation NHS Trust

## Abstract

**Aims:**

Healthcare triage for those subject to section 136 powers (MHA 1983/2007) remains challenging. Camden and Islington NHS Foundation Trust opened a dedicated Health-Based Place of Safety (HBPOS) in 2020, situated separately from an emergency department (ED). There was concern that this may lead to physical health problems going unrecognised. We aimed to design a simple, efficient algorithm to be used by non-medically-trained staff to identify those who are subject to s.136 powers who would benefit from medical clearance before being admitted to the HBPOS

**Method:**

We chaired a consensus meeting with nursing staff, police and emergency medicine consultants when designing the algorithm. Case notes of those presenting under s.136 to the POS over 1 calendar-month in 2019 were reviewed, and the proportion of those who the algorithm would have diverted for medical clearance was calculated. We then reviewed the proportion of cases sent for medical clearance during a single calendar month in 2020, after the HBPOS had opened, to see whether there was a significant difference.

**Result:**

37 patients were admitted to the ED-based POS in July 2019, of which 36 records were analysed. 9 patients (25%) were referred for medical clearance, with 2 (6%) requiring medical admission. 8.6% were identified as needing medical clearance when the algorithm was applied retrospectively (positive predictive value 66%, negative predictive value = 79%).

Review of records over 1 calendar-month after the HBPOS was established showed 30.6% of patients had been diverted for medical clearance prior to entering the HBPOS. Of the 65 patients, 1 (2%) required transfer to ED within 48 hours of entry. No statistical difference in the proportion of patients sent for medical clearance was observed since the formation of the HBPOS away from the ED (Chi-squared = 0.549, p = 0.458), suggesting the algorithm successfully identified those patients who needed medical clearance prior to admission.

We observed high rates of intoxication amongst those admitted (30–40%).

**Conclusion:**

The algorithm showed high specificity and negative predictive value, allowing for a degree of confidence when admitting those deemed at low-risk of physical deterioration, though it does not eliminate the need for clinical judgement. Interpretation of the results is complicated by the COVID19 pandemic in 2020, which was not accounted for in the algorithm, which possibly led to deviations from the algorithm in real-world clinical practice.

